# Changes of Visual Pathway and Brain Connectivity in Glaucoma: A Systematic Review

**DOI:** 10.3389/fnins.2018.00363

**Published:** 2018-05-29

**Authors:** Raffaele Nuzzi, Laura Dallorto, Teresa Rolle

**Affiliations:** Eye Clinic, Department of Surgical Sciences, University of Torino, Turin, Italy

**Keywords:** glaucoma, retinal ganglion cells, neurodegeneration, neuroplasticity, systematic review

## Abstract

**Background:** Glaucoma is a leading cause of irreversible blindness worldwide. The increasing interest in the involvement of the cortical visual pathway in glaucomatous patients is due to the implications in recent therapies, such as neuroprotection and neuroregeneration.

**Objective:** In this review, we outline the current understanding of brain structural, functional, and metabolic changes detected with the modern techniques of neuroimaging in glaucomatous subjects.

**Methods:** We screened MEDLINE, EMBASE, CINAHL, CENTRAL, LILACS, Trip Database, and NICE for original contributions published until 31 October 2017. Studies with at least six patients affected by any type of glaucoma were considered. We included studies using the following neuroimaging techniques: functional Magnetic Resonance Imaging (fMRI), resting-state fMRI (rs-fMRI), magnetic resonance spectroscopy (MRS), voxel- based Morphometry (VBM), surface-based Morphometry (SBM), diffusion tensor MRI (DTI).

**Results:** Over a total of 1,901 studies, 56 case series with a total of 2,381 patients were included. Evidence of neurodegenerative process in glaucomatous patients was found both within and beyond the visual system. Structural alterations in visual cortex (mainly reduced cortex thickness and volume) have been demonstrated with SBM and VBM; these changes were not limited to primary visual cortex but also involved association visual areas. Other brain regions, associated with visual function, demonstrated a certain grade of increased or decreased gray matter volume. Functional and metabolic abnormalities resulted within primary visual cortex in all studies with fMRI and MRS. Studies with rs-fMRI found disrupted connectivity between the primary and higher visual cortex and between visual cortex and associative visual areas in the task-free state of glaucomatous patients.

**Conclusions:** This review contributes to the better understanding of brain abnormalities in glaucoma. It may stimulate further speculation about brain plasticity at a later age and therapeutic strategies, such as the prevention of cortical degeneration in patients with glaucoma. Structural, functional, and metabolic neuroimaging methods provided evidence of changes throughout the visual pathway in glaucomatous patients. Other brain areas, not directly involved in the processing of visual information, also showed alterations.

## Introduction

### Rationale

Glaucoma is a leading cause of irreversible blindness worldwide (Tham et al., 2014) and is characterized by the death of retinal ganglion cells (RGC) and their axons (Weinreb et al., 2014). The global incidence of primary open angle glaucoma (POAG) is anticipated to increase to 65 million by 2020 (Kapetanakis et al., 2016).

Numerous studies showed brain changes, especially in the visual pathway in glaucoma (Gupta and Yücel, 2003; Davis et al., 2016). The neurodegenerative process has been established in glaucomatous damage (Gupta et al., 2007; Chang and Goldberg, 2012). As a matter of fact, POAG presents important analogies with other neurodegenerative diseases: it has been shown that RGC share the same cell death mechanisms with Alzheimer's disease, AD (McKinnon, 2012). Protein misfolding is one of the identified mechanisms capable of triggering the apoptotic cascade in glaucomatous pathogenesis (Wostyn et al., 2009). Indeed, the β-amyloid deposits, characteristic of Alzheimer's disease, have recently been implicated in the pathogenesis of glaucoma (Wostyn et al., 2010). The incidence of glaucoma is increased in patients with AD compared to controls with the same age (Bayer et al., 2002; Tamura et al., 2006). Furthermore, the progression of visual field defects is accelerated in patients with open-angle glaucoma and AD compared to patients with open-angle glaucoma without AD (Bayer and Ferrari, 2002).

Moreover, nowadays, new treatments targeted visual pathway. Several neuroprotective strategies and drugs have been studied and some of them are used in the clinical practice. Neuroprotection consist in the prevention of neurons death (Jutley et al., 2017; Sena and Lindsley, 2017). Molecules that have passed through clinical test are memantine (NMDA glutamate receptor antagonist) and brimonidine (alpha2-adrenergic agonist). Neuroprotection is not effective for RGCs which have already been injured, thus neuroenhancement is also proposed as a therapeutic approach. Axon regeneration is the aim of neurotrophic factors such as nerve growth factor (NGF) and ciliary neurotrophic factor (CNTF). Human trials using these exogenous neurotrophic factors showed promising results but of limited duration and with the inconvenient of repeated injections. In recent years, stem cells have been widely studied as potential source of cell replacement. Several types and methods of administration (subtenionan, retrobulbar, and intravitreal) have been proposed and experimented in clinical trials even in humans. Another approach, still limited to murine examples, is the RGC transplantation. Preliminary data showed promising approach in retinas with degenerating RGCs (Venugopalan et al., 2016). Finally, retinal implants provide electrical stimulation of different targets but their clinical application is limited due to limited visual perception (Mathieson et al., 2012).

The efficacy of these modern approaches impose a deep knowledge of, cortical reorganization, neuroplasticity, and rearrangement of the visual pathways in glaucoma. Almost all approaches targeted RGCs of optic nerve require the integrity of posterior visual pathway or the adaptation of nervous system through plastic capacity. Moreover, new insights into brain changes in glaucoma may stimulate new therapeutic strategies.

The advent of non-invasive brain-imaging techniques has led to a rapid growth in studies investigating the brain damage in glaucomatous patients. Newer techniques and protocols enable the study of anatomical gray and white matter changes through structural techniques, brain connectivity and functional responses after stimulation through functional magnetic resonance imaging (fMRI) and changes in brain metabolite levels through metabolic technique (Fiedorowicz et al., 2011; Mastropasqua et al., 2015; Brown et al., 2016; Prins et al., 2016).

Structural brain techniques include diffusion MRI (diffusion tensor imaging, DTI) which can reveal abnormalities in white matter structure and brain connectivity through diffusivity of water molecules along the axons (Alexander et al., 2007). Voxel-based morphometry analysis (VBM) which is computed on MRI, quantifies the tissue concentration of gray and white matter volume (Ashburner and Friston, 2000). Surface-based morphometry (SBM), throughT1-weighted morphometric analysis, provides data about the brain structure, such as thickness, curvature, and surface area of brain cortex (Clarkson et al., 2011).

Functional magnetic resonance imaging (fMRI) is a neuroimaging procedure capable to detect functional brain activities after a stimulus through detecting changes in blood flow (Miki et al., 2001, 2002; Kollias, 2004). It is also used to evaluate interactions between brain areas in a resting subject without any visual stimulus, the so-called “resting state fMRI” (Smitha et al., 2017). Finally, metabolic method such as the proton magnetic resonance spectroscopy (MRS) is able to detect and quantify certain biochemical compounds in brain tissue (Boucard et al., 2007).

### Objective

This systematic review focuses on brain changes detected with the modern techniques of neuroimaging (structural, functional, and metabolic methods) in glaucomatous subjects.

### Research question

What have studies on neuroimaging in glaucomatous patients found thus far?

When do brain changes occur and what is the cause of these changes in the glaucomatous patients?

## Methods

We adopted the Preferred Items for Systematic Reviews and Meta-Analyses (PRISMA) guidelines.

### Search strategy

We searched: MEDLINE (Ovid), CENTRAL (which contains the Cochrane Eyes and Vision Group Trials Register), EMBASE (Ovid), Latin American and Caribbean Literature on Health Sciences (LILACS), CINAHL (EBSCO), Trip Database, and The National Institute for Health and Care Excellence (NICE). The construction of search strategies was performed using database specific subject headings and keywords. The MEDLINE search strategy was provided as Supplementary Material (Supplementary Data 1). These searches were supplemented by hand searching the bibliographies of all the included studies.

Gray literature was not considered. Accepted languages of publication were: English, German, French, Spanish, Portuguese and Italian. Articles published until October 31, 2017 were included.

### Study design

Randomized controlled trials (RCT), clinical trials, non-randomized comparative studies, cohort studies, and case series (CS) were included.

Case report, case series with <6 patients and article with absence of outcome data were excluded.

### Participants, interventions, comparators

We included studies on patients affected by POAG, primary angle closure glaucoma (PACG), and normal tension glaucoma (NTG).

Glaucomatous damage could be both unilateral and bilateral. Stages of glaucoma were not exclusion criteria.

We included studies using the following neuroimaging techniques:

- Diffusion tensor MRI (DT MRI/ DTI)
- Voxel- based Morphometry (VBM)
- Surface-based Morphometry (SBM)
- Functional Magnetic Resonance Imaging (fMRI)
- Resting-state fMRI (rs-fMRI)
- Magnetic resonance spectroscopy (MRS)

### Data sources, studies sections, and data extraction

According to the PRISMA flow diagram screening of titles and abstracts was carried out. Not pertinent articles were rejected. Duplicates were removed using EPPI reviewer (by EPPI-Center, Social Science Research Unit, the Institute of Education, the University of London, UK). After this initial selection, full texts were independently judged for eligibility.

### Data analysis

The main outcome of this systematic review was the current understanding of brain changes in glaucomatous subjects. Structural, functional, and metabolic neuroimaging results were separately analyzed and presented, but their interaction was nonetheless studied. Differences between various types of glaucoma and various stages of disease were analyzed.

Specifically, characterizing parameters of each neuroimaging method are listed below:

- DTI: fractional anisotropy (FA), mean diffusivity (MD), and radial diffusivity (RD). They all are measures of white matter damage.
- VBM: gray matter and white matter volume.
- SBM: thickness, curvature, and surface area of brain cortex.
- fMRI: Blood oxygenation level dependent (BOLD) evaluation. It detects changes in the blood flow of local brain regions through changes in hemoglobin and unoxygenated hemoglobin in the blood. In the manuscript we referred to fMRI for “task-fMRI,” which measures responses during visual tasking
- rs-fMRI: Voxel-wise degree centrality (DC), that is the direct connections for a given voxel in the voxel-wise connectome, and functional connectivity (FC) between areas in resting conditions, without any visual tasks
- MRS: concentrations of the metabolites such as N-acetylaspartate (NAA), creatinine (Cr), and Choline (Cho)

## Results

### Study selection

**Figure 1:**
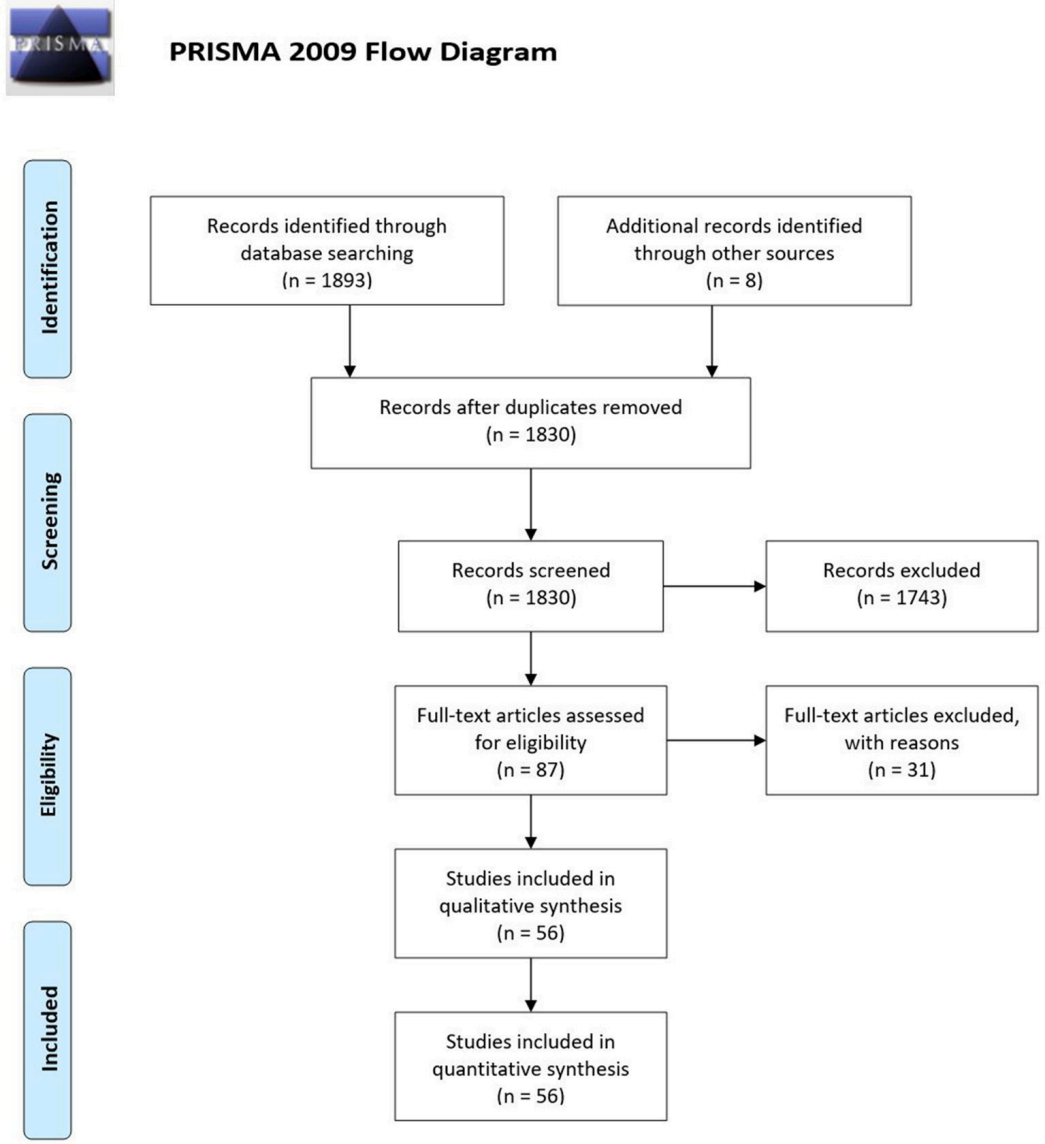
Prisma flow diagram.

A total of 1,901 studies were screened using the described search strategy. At the end of the selection process, 56 case series were included in the systematic review. Prisma flow diagram (Figure 1) gives details on screening process.

Thirty-one out of 56 (55%) studies examined brain changes in terms of structural parameters (19 studies used DTI, 6 studies VBM, 4 studies SBM, one single study both VBM and SBM, and one single study both VBM and DTI). Nineteen out of fifty-six studies, (34%) used functional techniques to evaluate brain changes following glaucomatous degeneration (10 studies used the fMRI and 9 studies the rs-fMRI). Metabolic methods (MRS) are used in 2 studies (4%). Four studies (7%) used both structural and functional techniques on the same group of patients: three studies used rs-fMRI, VBM, and DTI, one study used VBM and fMRI.

### Study characteristics

A total of 1,326 patient and 1,055 controls were considered in this systematic review. Forty-nine studies included POAG patient (41 studies only POAG patients, 7 studies included both POAG and NTG, one single study had both POAG and PACG). A total of 1,061 patient, corresponding to 80% of the total number had POAG. 154 out of 1326 (12%) patients were affected by PACG (they are included in seven studies, one of these studies included both POAG and PACG patients). NTG was the less studied type, corresponding to 8% (111 patients) of the total. Eight studies included NTG patients, 7 studies had both NTG and POAG patients, one single study had NTG patients).

Patients characteristics of included studies are reported in Table 1. Patients included in this review show high heterogeneity in mean age ranging from 25 (Dai et al., 2013) to 76 (Bogorodzki et al., 2014) years. Severity of glaucoma damage varies from early to severe and some authors included different stages in the same study. Method of severity classification was not consistent throughout all studies, even though the most used is the Hodapp–Parrish–Anderson (HPA) classification.

**Table 1:** Demographic and clinical characteristics.

**Author, year**	**G type**	**N patients**		**Age (years) Mean** ± **SD**		**Sex M/F (%)**		**Clinical characteristics**
		**G**	**H**	**G**	**H**	**G**	**H**	
Bogorodzki et al., 2014	POAG	14	12	76 ± 7.5	66.5 ± 9.8	57/43	25/75	Advanced unilateral POAG (no light perception to hand movement, CDR 0.9–1), fellow eye affected less than MD > −20 dB
Bolacchi et al., 2012	POAG	24	15	59 (M) 55 (F)	62 (M) 60 (F)	79/21	80/20	POAG patients are divided in early (stage 1 and 2 of Hodapp classification) and severe (stage 4 and 5) stage
Borges et al., 2015	POAG	9	4	70 ± 8.7	59 ± 8.2	89/11	75/25	Unilateral POAG (at least MD < −10 dB, paracentral scotoma of diameter > 10° within the central 20°) for at least 3 yrs
Boucard et al., 2007	POAG	7	12	73	62	86/14	67/33	Homonymous scotoma > 10° for at least 3 yrs. A group of 7 patients with AMD was also considered
Boucard et al., 2009	POAG	8	12	72	66	88/12	75/25	Homonymous scotoma > 10° for at least 3 yrs. A group of 9 patients with AMD was also considered
Boucard et al., 2016	NTG	30	21	52 ± 10.7	52.3 ± 15.3	77/23	48/52	In glaucomatous eyes, mean MD RE = −8.9 dB, LE = −6.8 dB
Cai et al., 2015	PACG	23	23	49.5 ± 14.4	48.2 ± 9.4	35/65	35/65	PACG eyes mean IOP = 39 mmHg. All patients underwent glaucoma surgery. 9/23 underwent MRI after surgery
Chen Z. et al., 2013	POAG	25	24	34.5	33.6	76/24	75/25	POAG subjects were divided on the basis of visual field severity in 6 groups
Chen W. W. et al., 2013	POAG	15	15	43.3 ± 4.1	43.9 ± 3.8	60/30	60/30	Bilateral advanced POAG (CDR > 0.9, MD < −15 dB).
Chen et al., 2017	PACG	20	20	54.4 ± 9.5	53.8 ± 9.2	50/50	50/50	NS
Dai et al., 2013	POAG	22	22	25	36	77/23	77/23	NS
Duncan et al., 2007a	POAG	6^*^	0	69.3 ± 7.7	NA	50/50	NA	Asymmetric POAG with one glaucomatous eye and a less affected controlateral eye
Duncan et al., 2007b	POAG	6^*^	0	69.3 ± 7.7	NA	50/50	NA	Asymmetric POAG with one glaucomatous eye and a less affected controlateral eye
El-Rafei et al., 2011	POAG—NTG	13 (7 POAG, 6 NTG)	10	64.7 ± 11.5	62.8 ± 13.6	46/54	30/70	NS
El-Rafei et al., 2013	POAG—NTG	57 (39 POAG, 18 NTG)	27	61.9 ± 8.6	58.5 ± 10.1	44/56	37/63	NS
Engelhorn et al., 2011	POAG	50	50	52.2 ± 12.6 (M) 60.0 ± 16.9 (F)	54.0 ± 14.2 (M) 61.4 ± 15.1 (F)	36/46	44/56	NS
Frezzotti et al., 2014	POAG	13	12	51.7 ± 6.6	47.3 ± 5.1	77/23	33/64	All patients had bilateral advanced glaucomatous visual field damage according with the Hodapp/Bascom Palmer classification (mean MD worse eye = −23.2 dB)
Frezzotti et al., 2016	POAG	57	29	62.1 ± 69.3	58 ± 10	67/23	52/48	POAG patients were classified according to the Hodapp/Bascom Palmer classification. 14 patients had early (Stage1), 13 moderate (Stage 2), and 30 severe (Stage 3) disease
Garaci et al., 2009	POAG	16	10	63	61	56/44	60/40	The glaucomatous eyes were stratified according to severity of visual field defects into six groups by using the Hodapp-Parrish system
Gerente et al., 2015	POAG	17	8	61.8 ± 10.9	56.4 ± 13.9	41/59	63/37	The patients were assigned to three subgroups: (Tham et al., 2014) initial glaucoma, (2) asymmetrical glaucoma and (3) severe glaucoma, according to the VF defect pattern in both eyes.
Giorgio et al., 2018	POAG—NTG	34 (17 POAG, 17 NTG)	29	58.6 ± 13.4(POAG) 58.9 ± 13.7(NTG)	57.9 ± 9.9	76/24 (NTG) 53/47 (POAG)	52/48	Glaucoma patients were classified with Hodapp/Bascom Palmer glaucoma severity Staging. Mild/moderate/severe = 10/2/5 in both POAG and NTG.
Hernowo et al., 2011	POAG	8	12	72	67	88/12	75/25	Participant inclusion criteria were the following: (1) a glaucomatous VF defect of at least 10° in diameter in at least one quadrant, affecting both eyes; (2) VF defects had to include the paracentral regions in both eyes; (3) the defects had to have been present for at least 3 years
Huang et al., 2015	PACG	21	21	52 ± 13.8	60.6 ± 4.6	38/62	43/57	NS
Jiang et al., 2017	POAG	13	13	32.4 ± 6.2	30.4 ± 4.8	77/23	77/23	Only early and mid-stage were included (stage 1, 2 and 3 of the Glaucoma Staging System 2)
Kaushik et al., 2014	POAG	9	9	69	68	44/56	33/67	Glaucoma patients with binocular, symmetrical superior, or inferior visual hemifield defects were selected. The difference between the unaffected and affected hemifield sensitivity (in decibels) had to be more than 3:1.
Lestak et al., 2014	POAG—NTG	16 (8 POAG, 8 NTG)	8	Range 40–73 (POAG) 40–70 (NTG)	23-65	63/27	25/75	NS
Li et al., 2012	POAG	30	30	50	NS	80/20	80/20	According to Becker visual field stages POAG patients were divided into 9 early stage and 21 advanced-late stage.
Li et al., 2014	POAG	21	22	46.4 ± 16.4	45.6 ± 11.9	48/52	50/50	NS
Li et al., 2017	PACG	25	25	52	52	40/60	40/60	PACG eyes mean IOP = 31 mmHg. All patients underwent glaucoma surgery. 19/25 underwent MRI after surgery
Lu et al., 2013	POAG	15	15	48 ± 20	48 ± 19	93/7	93/7	NS
Michelson et al., 2013	POAG-NTG	26 (13 POAG, 13 NTG)	7	57.4 ± 12.7 (POAG) 59.5 ± 13.1 (NTG)	54.6 ± 12.1	46/54 (POAG) 38/62 (NTG)	43/57	Glaucoma severity were assessed with HRT
Murai et al., 2015	POAG	32	19	59.5 ± 13.7	56.5 ± 14	53/47	63/37	Average MD −7.17 dB in the right eye and −8.26 dB in the left eye.
Murphy et al., 2016	POAG	26 (13 early−13 advanced)	9	62.4 ± 2.1 (early) 63.6 ± 2.3 (advanced)	61.3 ± 3.1	54/46 (early) 39/61 (advanced)	33/67	NS
Nucci et al., 2012	POAG	24	12	60	NS	70/30	NS	NS
Omodaka et al., 2014	POAG	19	0	66.1 ± 9.0	NA	68/32	NA	16 eyes were POAG and 22 were NTG
Qing et al., 2010	POAG	6	0	49.5	NA	33/67	NA	Asymmetric visual field damage and spared central vision.
Schoemann et al., 2014	POAG	39	22	63.9 ± 9.3	63.3 ± 11.9	33/67	41/59	NS
Sidek et al., 2014	POAG	60 (30 mild, 30 severe)	30	68.4 ± 8.9 (mild) 65.6 ± 9.0 (severe)	63.5 ± 8.4	37/67 (mild) 73/27 (severe)	40/60	The categorisation into mild and severe glaucoma was done using the Hodapp–Parrish–Anderson (HPA) classification.
Song et al., 2012	PACG	30	16	61 ± 8 L stimulation 61 ± 8 (R stimulation)	57 ± 8	37/63	38/62	The patients were assigned to the early VF defect group (paracentral scotoma, nasal ladder) or the advanced VF defect group (quadrantanopia, central visual field and temporal island).
Song et al., 2014	POAG	39	41	34.8 ± 9.9	34.8 ± 9.7	82/18	81/19	NS
Tellouck et al., 2016	POAG	50	50	61.9 ± 6.9	61.9 ± 7.0	40/60	40/60	Severity of glaucoma was defined on Hodapp-Parrish-Anderson classification
Wang et al., 2013	PACG	23	20	54	50	39/61	35/65	Severity of glaucoma was defined on Hodapp-Parrish-Anderson classification
Wang et al., 2016	POAG	25	25	44.6 ± 13.0	36.8 ± 11.6	44/56	52/48	NS
Wang et al., 2016a	POAG	25	25	44.6 ± 13.0	36.8 ± 11.6	44/56	52/48	NS
Wang et al., 2016b	POAG	25	25	44.6 ± 13.0	36.8 ± 11.6	44/56	52/48	NS
Williams et al., 2013	POAG	15	15	66.1 ± 11.2	65.6 ± 11.3	67/33	67/33	NS
Yu et al., 2013	POAG	36	40	46.5	46.5	75/25	74/26	Two subgroups (a mild and severe group) based on Hodapp-Parrish-Anderson classification
Yu et al., 2014	POAG	36 (19 + 17)	20	42.6 ± 14.5 (mild) 48.1 ± 16.6 (severe)	43.3 ± 15.1	57/43 (mild) 71/29 (severe)	55/45	19 mild and 17 severe POAG patients based on Hodapp-Parrish-Anderson classification
Yu et al., 2015	POAG	37 (20 + 17)	20	43.6 ± 14.5 (mild) 48.1 ± 16.6 (severe)	43.3 ± 15.1	59/41 (mild) 71/29 (severe)	55/45	20 mild and 17 severe POAG patients based on Hodapp-Parrish-Anderson classification
Zhang et al., 2012	NTG	30	30	54.8 ± 11.9	53.9 ± 11.2	50/50	53/47	NS
Zhang et al., 2013	POAG-PACG	20	20	45	45	40/60	40/60	8 POAG, 12 PACG. Different stages (from early to advanced)
Zhang et al., 2015	POAG	23	29	47 ± 7	48 ± 7	61/39	66/36	9 early-moderate glaucoma and 14 advanced glaucoma
Zhang et al., 2016	POAG—NTG	18 (10 + 8)	18	33.0 ± 5.6	33.0 ± 5.6	78/22	78/22	10 POAG, 8 NTG all early stage
Zhou et al., 2017a	POAG	11	11	60.0 ± 9.2	55.9 ± 7.5	36/64	64/34	Mild to moderate POAG
Zhou et al., 2017b	POAG	9	9	61 ± 11	58 ± 5	44/56	67/33	NS
Zikou et al., 2012	POAG	18	18	57.1 ± 11.4	NS	78/22	NS	NS

* *Duncan et al. (2007a) and Duncan et al. (2007b) share the same patients, G, glaucoma; H, healthy; CDR, Cup/disc ratio; yrs, years; NR, not reported; MD, Mean Deviation; RE, right eye; LE, left eye; RNFL, retinal nerve fiber layer; NS, not specified; NA, not applicable; VF, visual field; HRT, Heidelberg Retina tomograph*.

### Outcomes of structural analysis

Results of studies analyzing structural brain changes in glaucomatous patients are reported in Table 2.

**Table 2:** Outcomes of studies with structural brain analysis.

**Author, year**	**Brain area analyzed**	**Parameter analyzed**	**Results**
**DTI**			
Bolacchi et al., 2012	Intraorbital optic nerve at two different levels: proximal and distal to the ONH	Fractional anisotropy (FA) and mean diffusivity (MD)	At early stage higher MD at the proximal site with respect to the distal site. In contrast, at severe stages both the proximal and the distal portion showed altered MD. FA is altered in both stages both in the proximal and distal part.
Boucard et al., 2016	White matter of all brain	FA and MD	Reduced FA in clusters in bilateral occipital pole (comprising OR and forceps major), superior parietal lobe, body and splenium of corpus callosum.
Chen Z. et al., 2013	White matter of all brain	FA and MD. Correlation with clinical measurement	Bilateral OT and OR showed decreased FA and increased MD. FA correlates with CDR, RNFL thickness and visual function.
El-Rafei et al., 2011	Optic radiation	Axial, radial, mean diffusivities and FA	Glaucoma subjects have increased radial diffusivity and mean diffusivity significant voxels with a main concentration in the proximal part of the right optic radiation
El-Rafei et al., 2013	A specified ROI on the segmented optic radiation	Different diffusion tensor derived measures. Their ability for detecting and discriminating different glaucoma entities	The discrimination accuracy between healthy and glaucoma (POAG and NTG) subjects was 94.1%, between healthy and POAG was 92.4% and it increased to 100% between healthy and NTG groups. Discrimination between glaucoma entities (POAG and NTG) had an accuracy of 98.3%.
Engelhorn et al., 2011	Optic radiation	Volume of the optic radiation and grading of microangiopathic lesions (mild, moderate and severe)	44% glaucoma patients showed significant rarefaction of the optic radiation: the volume was reduced to 67 ± 16% compared with controls. Cerebral microangiopathy of OR was higher among glaucoma patients.
Frezzotti et al., 2014	Whole brain white matter	FA, axial diffusivity (AD), radial diffusivity (RD)	Altered integrity (decreased FA or increased diffusivities) along the visual pathway (optic tracts, chiasm, radiation) of POAG and also in nonvisual WM tracts (superior longitudinal fascicle, anterior thalamic radiation, corticospinal tract, middle cerebellar peduncle).
Frezzotti et al., 2016	Whole brain white matter	FA, AD, RD. Differences between healthy and whole POAG and between healthy and early stage POAG.	Decreased FA and higher AD along the visual pathway (optic tracts, chiasm, radiation) of POAG and also in nonvisual WM tracts (superior longitudinal fascicle, supramarginal gyrus and superior parietal lobule). Similar results in the early stage glaucoma.
Garaci et al., 2009	Optic nerve and optic radiation	FA and MD. Correlation with disease severity	POAG NO and OR had significantly higher MD and significantly lower FA. A negative correlation between mean FA for the optic nerves and glaucoma stage was observed.
Giorgio et al., 2018	Whole brain white matter	FA, AD, RD. Differences between healthy, POAG and NTG.	Decreased FA and higher AD along the visual pathway (optic tracts, chiasm, radiation) and also in nonvisual WM tracts (superior longitudinal fascicle, WM adjacent to precuneus, inferior frontal gyrus, superior parietal lobe) in both POAG and NTG. Differences were found in the nonvisual areas abnormalities between POAG and NTG.
Kaushik et al., 2014	Optic radiation	RD, AD, MD, FA in OR. OR fibers were separated into tracts subserving the superior of inferior hemifield of the visual field.	FA was lower and MD was higher in POAG OR compared with controls. Unaffected OR tracts showed changes in RD compared with controls
Lu et al., 2013	Occipital white matter	FA	Occipital white matter in POAG had lower FA values.
Michelson et al., 2013	Optic radiation	FA, AD and RD of the optic radiations and their correlation with glaucoma severity indicators	DTI-derived parameters of the axonal integrity (FA, AD) and demyelination (RD) of the optic radiation are linked to HRT-based indices of glaucoma severity.
Murai et al., 2015	Optic radiation	FA of optic radiation. Correlation with glucose metabolism in the striate cortex studied with PET	FA in optic radiations was lower in patients with glaucoma. There were significant correlations between FA of the optic radiation and ipsilateral striatal glucose metabolism.
Nucci et al., 2012	Optic nerve	FA and MD. Correlation with optic nerve structure (GDx-VCC, HRT, OCT)	DTI parameters of the axonal architecture of the optic nerve show good correlation with morphological features of the optic nerve head and RNFL documented with GDx-VCC, HRT-III and OCT.
Omodaka et al., 2014	Optic nerve	FA and AD. Correlation with optic nerve structure (OCT) and MD	DTI parameters correlated with RNFL and MD at VF.
Schoemann et al., 2014	Optic radiation	FA. Correlation with the extent of cerebral white matter lesions (WML).	There was a significant correlation between FA and WML in POAG regarding the total brain, the periventricular region, and the optic radiation in both hemispheres.
Sidek et al., 2014	Optic nerve and optic radiation	FA and MD and their discriminant power between mild and severe glaucoma and correlation with RNFL.	FA and MD in the optic nerve and optic radiation decreased and increased respectively as the disease progressed. FA at the optic nerve had the highest sensitivity (87%) and specificity (80%). FA values displayed the strongest correlation with RNFL thickness in the optic nerve.
Tellouck et al., 2016	Optic radiations	FA, MD, RD, AD	FA was lower and RD higher than controls. Correlation with functional and structural damage.
Wang et al., 2013	Optic nerve	FA, MD. Correlation with RNFL measured with OCT	FA was lower and MD higher than controls. Correlation with RNFL.
Zhang et al., 2012	Optic nerve	FA, MD. Correlation with RNFL measured with OCT	FA was lower and MD higher than controls. Correlation with RNFL.
Zhou et al., 2017a	Optic tract and optic radiation	FA, RD, AD, MD. Correlation with visual field loss and RNFL	FA was lower along the optic tracts and radiations in POAG. FA correlated with visual field loss but not with RNFL.
Zikou et al., 2012	Whole brain white matter	FA and MD	A significant decrease of FA was observed in the inferior fronto-occipital fasciculus, the longitudinal and inferior frontal fasciculi, the putamen, the caudate nucleus, the anterior and posterior thalamic radiations, and the anterior and posterior limbs of the internal capsule of the left hemisphere.
**VBM**			
Boucard et al., 2009	whole brain 21 mm diameter VOI at the posterior pole: 1 posterior and 1 anterior in both superior and inferior banks of the calcarine sulcus in each hemisphere	Changes in gray matter density in the all brain and VOI analysis.	(1) bilateral reduction of gray matter density on the medial aspect of the occipital lobe, at the anterior half of the calcarine fissures. (2) gray matter density is more reduced in the anterior than in the posterior region.
Chen Z. et al., 2013	Whole brain	Differences in gray matter volume (GMV)	POAG showed a significantly decreased GMV in the lingual gyrus, calcarine gyrus, postcentral gyrus, superior frontal gyrus, inferior frontal gyrus, and rolandic operculum of both sides, and in the R inferior occipital gyrus, L paracentral lobule, R supramarginal gyrus, and R cuneus. The GMV was significantly larger in POAG in both sides of the middle temporal gyrus, inferior parietal gyrus, angular gyrus, and L superior parietal gyrus, L precuneus, and L middle occipital gyrus.
Frezzotti et al., 2014	Whole brain	Differences in gray matter volume	POAG patients showed brain atrophy in both visual cortex and other distant GM regions (frontoparietal cortex, hippocampi and cerebellar cortex).
Frezzotti et al., 2016	Whole brain	Differences in gray matter volume	No differences considering whole POAG. Lower GM volume in occipital cortex and hippocampus only in advanced POAG.
Giorgio et al., 2018	Whole brain	Differences in gray matter volume between healthy, POAG and NTG.	Both groups showed reduced GMV in visual cortex and beyond it. Compared with NTG, POAG had more atrophic visual cortex.
Hernowo et al., 2011	ROI: optic nerve, optic chiasm, optic tracts, lateral geniculate nuclei (LGN) and the optic radiations	Volume of ROIs and correlation with VF sensitivity	Compared with the controls, subjects with glaucoma showed reduced volume of all structures along the visual pathway, including the optic nerves, the optic chiasm, the optic tracts, the LGN, and the optic radiations. No significant correlation between the volume of visual pathway and MD of VF.
Jiang et al., 2017	Whole brain	Differences in gray matter volume. Correlation with RNFL	The regions of the brain with increased volumes compared with the control group were the midbrain, L brainstem, frontal gyrus, cerebellar vermis, L inferior parietal lobule, frontal lobe, caudate nucleus, thalamus, precuneus, and BA 7, 18, and 46.
Li et al., 2012	Whole brain	Differences in gray matter volume	Compared with controls, brain regions with GM density changes were not found in the early stage but only in the advanced-late stage of POAG patients. GM density reduction was mainly located in the bilateral primary visual cortex (BA17 and BA18), bilateral paracentral lobule (BA5), R precentral gyrus (BA6), R middle frontal gyrus (BA9), R inferior temporal gyrus (BA20), R angular gyrus (BA39), L praecuneus (BA7), L middle temporal gyrus (BA21), and superior temporal gyrus (BA22). Increased GM density was found in BA39. In the advanced-late stage of POAG, some reduced GM density areas were related to binocular mean defect (MD) and disease duration.
Wang et al., 2016	Whole brain and ROI (LGN, V1, V2, amygdala, hippocampus)	Gray matter volume. Correlation with clinical parameters.	Significant volume shrinkages in the LGN bilaterally, R V1, L amygdala and no difference in V2 and hippocampus. Correlation with clinical variables.
Williams et al., 2013	93 brain structures	Differences in gray matter volume	5 differed regions differ significantly between POAG and healthy: all were larger in the glaucoma group and were all components of the visual association cortex. Total brain volume was also larger in the glaucoma group.
Zhang et al., 2015	Whole brain	Differences in gray matter volume	Compared to controls, a region with significant reduction of GMV was detected in the anterior calcarine fissure of advanced POAG patients but not in early-moderate POAG.
Zikou et al., 2012	Whole brain	Differences in gray matter volume	In POAG there were a significant reduction in the L visual cortex volume, the L lateral geniculate nucleus, and the intracranial part of the ONs and the chiasma.
**SBM**			
Bolacchi et al., 2012	Whole brain cortex	Cortical thickness on flat map	Local thinning in the visual cortex areas in POAG: 1 cluster in BA19 LH (lingula), 2 cluster in BA19 RH (fusiform gyrus and cuneus).
Wang et al., 2016	V1 and V2	Cortical thickness. Correlation with clinical parameters.	The right V1 thickness was significantly reduced in glaucoma. Correlation with clinical variables.
Yu et al., 2013	Whole brain cortex	Cortical thickness. Correlation with clinical parameters.	POAG patients showed bilateral cortical thinning in the anterior half of the visual cortex around the calcarine sulci (calcarine cortex) including the right BA 17 and left BA 17 and BA 18. Some smaller regions located in the left middle temporal gyrus (BA37) and the fusiform gyrus (BA19) also showed thinning relative to normal controls. The thickness of the VC correlated positively with RNFL thickness. Significant differences between mild and severe groups were observed.
Yu et al., 2014	ROIs: V5/MT+, anterior and posterior subregions of V1 and V2	Cortical thickness and volume in normal, mild (MP) and severe (SP) POAG patients. Correlation with clinical parameters	Decreased cortical thickness in V5/MT+ area in the MP group and in all of the visual areas except the posterior subregion of V1 in the SP group. Gray matter volume in the posterior subregion of V2 and mean curvature in the V5/MT+ were significantly changed in the SP group. The clinical measurements were positively correlated with the cortical thickness.
Yu et al., 2015	ROIs: V1, V2, ventral V3, V4 and V5/MT+	Cortical thickness in normal, mild (MP) and severe (SP) POAG patients. Correlation with clinical parameters	Decreased cortical thickness was detected in the bilateral V5/MT+ areas in the MP group and the L V1, bilateral V2 and V5/MT+ areas in the SP group. Cortical thinning of the bilateral V2 areas was detected in the SP group compared with the MP group. Cortical thinning of these visual areas was related to the ophthalmologic measurements.

*BA, Brodmann Area; LH, Left Hemisphere; RH, Right Hemisphere; VOI, Volume of interest; OR, optical radiation; OT, optic tracts; CDR, cup-disc ratio; RNFL, retinal nerve fiber layer; WM, white matter; GM, gray matter; GMV, gray matter volume; MD, mean deviation; MT+, middle temporal visual area*.

Diffusion tensor imaging methodology has been extensively used to investigate differences in white matter tracts of visual pathway within glaucoma (19 out of 56 studies, 34% of all included studies). Decreased fractional anisotropy (FA) and increased mean diffusivity (MD) and radial diffusivity (RD) are sign of axon injury. Differences of microstructure in white matter structures have been found in the optic nerve, optic tracts, optic chiasm, optic radiations, and occipital lobe. These results were obtained in studies including POAG (Garaci et al., 2009; El-Rafei et al., 2011, 2013; Engelhorn et al., 2011; Bolacchi et al., 2012; Nucci et al., 2012; Zhang et al., 2012; Zikou et al., 2012; Chen Z. et al., 2013; Lu et al., 2013; Michelson et al., 2013; Frezzotti et al., 2014, 2016; Kaushik et al., 2014; Omodaka et al., 2014; Schoemann et al., 2014; Sidek et al., 2014; Murai et al., 2015; Tellouck et al., 2016; Zhou et al., 2017a; Giorgio et al., 2018), NTG (Boucard et al., 2016) and PACG (Wang et al., 2013) patients.

Some of the studies using DTI methodology found alterations of white matter integrity not limited to the primary visual pathway. Boucard et al. (2016) revealed damage of corpus callosum and parietal lobe in NTG, Frezzotti et al. (2016) found alterations in the longitudinal fascicle, supramarginal gyrus, and superior parietal lobule of early stage open angle glaucoma. In addition, according to Giorgio et al study (Giorgio et al., 2018), superior longitudinal fascicle, white matter adjacent to precuneus, inferior frontal gyrus, and superior parietal lobe had microstructural alterations in both normal tension and open angle glaucoma compared to healthy subjects (Giorgio et al., 2018). Such white matter structures are related to higher aspects of visual and cognitive process. Furthermore, significant decreased FA was found in glaucomatous compared to healthy subjects in the inferior fronto-occipital fasciculus (implied in visuospatial function), longitudinal and inferior frontal fasciculi (related to visual memory), putamen, caudate nucleus, anterior, and posterior thalamic radiations and anterior and posterior limbs of the internal capsule (implied in direction, color, and orientation; Zikou et al., 2012).

Dependence on glaucoma severity of changes in white matter tissue studied with DTI was demonstrated. Glaucoma severity showed a negative correlation with FA, and a positive correlation with MD and RD values of white matter tracts involved in the visual process (Garaci et al., 2009; Nucci et al., 2012; Zhang et al., 2012; Chen Z. et al., 2013; Michelson et al., 2013; Wang et al., 2013; Omodaka et al., 2014; Sidek et al., 2014; Tellouck et al., 2016; Zhou et al., 2017a). Correlation was found between DTI parameters and RNFL, mean deviation of visual field, cup-to-disc ratio.

Significant gray matter reduction within the primary visual cortex emerged from many studies using VBM (Hernowo et al., 2011; Li et al., 2012; Zikou et al., 2012; Chen W. W. et al., 2013; Williams et al., 2013; Frezzotti et al., 2014, 2016; Zhang et al., 2015; Wang et al., 2016; Jiang et al., 2017; Giorgio et al., 2018). Specifically, a significant reduction was found only in the anterior part of occipital pole (Boucard et al., 2007) strengthen the idea of retinotopic damage. Other studies showed clusters of gray matter reduction in glaucoma patients in areas other than occipital pole, including the lingual gyrus, calcarine gyrus, postcentral gyrus, superior frontal gyrus, inferior frontal gyrus, rolandic operculum, cerebellar cortex, and hippocampi (Li et al., 2012; Chen W. W. et al., 2013; Frezzotti et al., 2014).

The extent of gray matter atrophy was dependent on the level of glaucoma severity (Li et al., 2012; Zhang et al., 2015; Wang et al., 2016). Indeed, Frezzotti et al found lower gray matter volume only in advanced POAG (Frezzotti et al., 2016).

Moreover, gray matter was increased in some brain areas (middle temporal gyrus, inferior parietal gyrus, angular gyrus, midbrain, brainstem, frontal gyrus, cerebellar vermis, thalamus) of glaucomatous patients (Li et al., 2012; Chen W. W. et al., 2013; Williams et al., 2013; Jiang et al., 2017). All these brain regions involved were associated with visual functions.

Alterations in visual cortex has been demonstrated also with SBM. In particular, the five studies using SBM demonstrated a thinning in the visual cortex of POAG (Bolacchi et al., 2012; Yu et al., 2013, 2014, 2015; Wang et al., 2016). Reduced cortex thickness was not limited to primary visual cortex, but also involved association visual areas, such as lingula, fusiform gyrus, cuneus, middle temporal gyrus (Bolacchi et al., 2012; Yu et al., 2013, 2014, 2015). Positive correlation was found between thinning of visual cortex and clinical measurements (Yu et al., 2013, 2014, 2015; Wang et al., 2016). Correlation with glaucoma severity was also demonstrated by significant differences of visual cortex thickness between mild and severe glaucoma groups, with increased damage in late-stage glaucoma (Yu et al., 2013).

### Outcomes of functional and metabolic analysis

Studies reporting functional and metabolic activity in glaucomatous patients are reported in Table 3.

**Table 3:** Outcomes of studies with functional and metabolic brain analysis.

**Author, year**	**Brain area analyzed**	**Parameter analyzed**	**Results**
**fMRI**			
Borges et al., 2015	V1 and V2 areas in both hemisphere	BOLD response within the LPZ and a matched area corresponding to healthy retina (control ROI) Stimulus: central filed (16°) dynamic checkerboard with high and low contrast monocularly presented	Reduction of activation in the LPZ compared to control ROI for stimulus in glaucomatous eye (only for medium and high contrast stimuli) in both V1 and V2. No differences in the fellow eye responses.
Duncan et al., 2007a	Visual areas (V1, V2, V3)	BOLD responses after stimuli are presented in the periphery. Different stimuli, all with contrast-reversing checkboard pattern: expanding rings, rotating wedges, meridian-mapping stimulus, 16° isopter, full-field contrast-reversing checkboard. Correlation with ON assessment	Reduced activity in V1. Pattern of deterioration of BOLD activity reflected pattern of deterioration of optic disc (evaluated with three techniques: OCT, HRT, GDx).
El-Rafei et al., 2013	Visual areas (V1, V2, V3)	BOLD responses after stimuli are presented in the periphery. Different stimuli, all with contrast-reversing checkboard pattern: expanding rings, rotating wedges, meridian-mapping stimulus, 16° isopter, full-field contrast-reversing checkboard. Correlation with visual filed defect	The spatial pattern of activity observed in V1 agreed with the pattern of visual field loss.
Gerente et al., 2015	Occipital pole and calcarine ROIs	BOLD responses to binocular stimuli (polar angle stimulus consisting of a rotating wedge). Association with structural and functional ocular findings.	Significant associations between binocular VF sensitivities and RNFL thickness with fMRI responses in the occipital pole and the calcarine ROIs.
Jiang et al., 2017	Whole brain	BOLD responses to 8 Hz black and white checkboard contrast stimuli	Higher brain activation in POAG was primarily located in the R supramarginal gyrus, frontal gyrus, superior frontal gyrus, L inferior parietal lobule, L cuneus, L midcingulate area and frontal lobe. Only the L cuneus negatively correlated with RNFL. R inferior parietal lobule, middle frontal gyrus, middle occipital gyrus, and inferior temporal gyrus showed positive correlations with RNFL.
Lestak et al., 2014	Visual cortex	BOLD responses to stimuli: black/white (BW) and yellow/blue (YB) checkerboard pattern	The extent of activation did not differ statistically between glaucomatous (both POAG and NTG) and controls. The difference in the magnitude of activation during the BW and YB stimulation is markedly higher in the POAG. No differences between BW and YB in NTG and controls.
Murphy et al., 2016	Visual cortex and higher-order visual brain	BOLD responses to 8 Hz Flickering stimuli	Reduced visual cortex activity. The primary visual cortex also exhibited more severe functional deficits than higher-order visual brain areas. The primary VC was reduced before visual field loss.
Qing et al., 2010	Visual cortex	BOLD responses to 8 Hz hemifield checkboard contrast stimuli	The BOLD fMRI signal change in the primary visual cortex corresponding to central visual input from the more severely affected eye was less than that of the fellow eye.
Song et al., 2012	Primary and Secondary visual cortex	BOLD responses to 8 Hz full-screen black and white flip checkboard stimuli.	The extent and intensity of visual cortex activation was decreased. In PACG patients.
Zhang et al., 2016	Different layers of the LGN, superior colliculus (SC), early visual cortices (V1, V2 and MT)	Responses to M stimulus (low spatial frequency at 30% luminance contrast, at 10 Hz) and P stimulus (high spatial frequency, isoluminant red/green square wave pattern, reversing contrast at 0.5 Hz)	Early glaucoma patients showed more reduction of response to transient achromatic stimuli than to sustained chromatic stimuli in the magnocellular layers of the LGN, as well as in the superficial layer of the SC. Magnocellular responses in the LGN were also significantly correlated with the degree of behavioral deficits to the glaucomatous eye. Early glaucoma patients showed no reduction of fMRI response in the early visual cortex.
Zhou et al., 2017b	Retinotopic areas (V1, V2, V3)	Cortical magnification factors and BOLD% changes as a function of eccentricity. 2 visual stimuli: a series of rotating wedges and a series of expanding or contracting rings. Correlation analysis between BOLD% changes and visual field scores, and between BOLD% changes and RNFL thicknesses	BOLD changes of POAG were reduced compared to normal. fMRI retinotopic mapping revealed enlarged representation of the parafovea in the visual cortex of POAG.
**rs-fMRI**			
Cai et al., 2015	Spontaneous brain functional connectivity within the whole brain	Voxel-wise degree centrality (DC) = direct connections for a given voxel in the voxel-wise connectome before and 3 months after surgery. Correlation of DC with clinical values	PACG pre-surgery: decreased DC in bilateral VC, increased DC in left ACC and caudate. PACG post-surgery: increased DC in bilateral VC and L precentral gyrus compared to pre-surgery. Negative correlation between DC in VC and IOP pre-surgery.
Chen et al., 2017	Spontaneous regional brain activity in the visual cortex	Intrinsic functional spontaneous neuronal activity thought regional homogeneity (ReHo). Correlation with clinical measurements	Compared with controls, PACG showed higher ReHo value in the L fusiform gyrus (BA37), L cerebellum anterior lobe, R frontal-temporal space (BA48), and R insula (BA48), and lower ReHo value in the bilateral middle occipital gyrus (BA18), L claustrum, and R paracentral lobule lobe (BA4). Significant correlation with duration disease, RNFL, CDR.
Dai et al., 2013	ROIs defined within Brodmann areas related to vision (BA17, BA18, BA 19, BA7)	Functional connectivity (FC) between the ROIs and other brain areas	Decreased FC in the POAG group between BA17 and R inferior temporal, L fusiform, L middle occipital, R superior occipital, L postcentral, R precentral gyri, and anterior lobe of the left cerebellum. Increased FC was found between BA17 and the L cerebellum, R middle cerebellar peduncle, R middle frontal gyrus, and extra-nuclear gyrus. Positive FC was disappeared between higher visual cortices (BA18/19) with the cerebellar vermis, R middle temporal, and R superior temporal gyri. Negative FC disappeared between BA18/19 and the R insular gyrus
Frezzotti et al., 2014	Spontaneous brain functional connectivity within the whole brain	Functional connectivity (FC) across 8 defined resting state networks (RSNs): visual, auditory, sensorimotor, default mode, working memory (right and left), dorsal attention and executive networks.	Decreased FC in visual, working memory and dorsal attention networks and increased FC in visual and executive networks.
Frezzotti et al., 2016	Spontaneous brain functional connectivity within the whole brain	Functional connectivity (FC) across 13 defined resting state networks (RSNs): visual (VN), auditory, sensorimotor, default mode (DMN, anterior and posterior), working memory (WMN, right and left), fronto-medial and orbitofrontal, executive control, salience, subcortical (ScN), temporal pole networks	POAG patients had lower FC in the VN and in the WMN, higher FC in the DMN and in the ScN. These abnormalities were already present in the subgroup of patients with stage 1.
Giorgio et al., 2018	Spontaneous brain functional connectivity within the whole brain	Functional connectivity (FC) across 12 defined resting state networks (RSNs): default mode, frontal executive control (ECN, medial, and medio-lateral), right and left frontoparietal working memory, dorsal attention, auditory/language ventral attention (VAN), visual (VN, primary, and secondary), medial temporal (limbic) and cerebellar networks	FC was altered in NTG at short-range level [visual network (VN), ventral attention network] and in POAG at long-range level (between secondary VN and limbic network). FC of POAG was higher than NTG in both VN and executive network.
Huang et al., 2015	Spontaneous brain functional connectivity within the whole brain	Amplitude of low-frequency fluctuation (ALFF), an index to detect spontaneous neuronal activity	Compared with healthy, PACG patients had significantly lower ALFF areas in the left precentral gyrus, bilateral middle frontal gyrus, bilateral superior frontal gyrus, right precuneus, and right angular gyrus, and higher ALFF area in the right precentral gyrus. There were significant negative correlations between the mean ALFF signal value of the middle frontal gyrus and the contralateral mean RNFL thickness.
Li et al., 2014	Spontaneous brain functional connectivity within the whole brain	ALFF. Correlation between ALFF and the disease stage	Compared with controls, POAG patients showed significantly decreased ALFF in the visual cortices, posterior regions of the default-mode network (DMN), and motor and sensory cortices. ALFFvalues were increased in the prefrontal cortex, L superior temporal gyrus (STG), R middle cingulate cortex (MCC), and Linferior parietal lobule (IPL). Severity disease stage correlated with ALFF of some areas (L cuneus, bilateral MTG and R prefrontal cortex).
Li et al., 2017	Intrinsic functional connectivity (iFC) in the centers of the V1	Seed-based iFC analysis before and 3 months after the surgery. Correlation between iFC and clinical variables (disease duration, IOP, RNFLT, CDR, VA)	*Pre-PACG*: compared to healthy, decreased iFC between L V1 and R V2 (covering the cuneus, calcarine and lingual gyrus) and increased iFC between L V1 and L temporal-parietal, L and R frontal opercula-insula-basal ganglia region, and R inferior parietal lobule. *Post-PACG*: increased iFC between the L V1 and bilateral V2, and between the L V1 and L or R postcentral gyrus; decreased iFC between the L V1 and the dorsal-attention and frontoparietal control networks. Correlation between iFC and VA
Song et al., 2014	Spontaneous regional brain activity in the visual cortex	Intrinsic functional spontaneous neuronal activity thought regional homogeneity (ReHo). Correlation with clinical parameters	Compared to controls, POAG showed increased ReHo in the R dorsal anterior cingulated cortex, the bilateral medial frontal gyrus and the R cerebellar anterior lobe, and decreased ReHo in the bilateral calcarine, precuneus gryus, pre/postcentral gyrus, L inferior parietal lobule and L cerebellum posterior lobe. Changes in spontaneous activity are associated with clinical parameters.
Wang et al., 2016a	Functional communication of anatomically separated structures.	Network analysis at both global and local levels	No significant differences of the global network measures were found between the two groups. However, the local measures were radically reorganized in glaucoma patients.
Wang et al., 2016b	Functional connectivity	Alterations of functional connectivity (FC) and connections within and between the subnetworks of the visual network and the default mode network (DMN) in glaucoma	FC analysis showed that the FC in the occipital pole of the visual network was decreased in POAG patients while no alterations were found in the FC of the DMN in patients.
**MRS**			
Boucard et al., 2007	Occipital pole in both hemisphere	Concentrations of the0 metabolites N-acetylaspartate (NAA), Creatine (Cr) and Choline (Cho)	No significant differences for any metabolites concentration between glaucoma, age-related macular degeneration and healthy groups.
Zhang et al., 2013	Geniculo-calcarine and the striate area of occipital lobe	Ratio of N-acetylaspartate (NAA)/Creatine (Cr), Choline (Cho)/Cr, glutamine and glutamate (Glx)/Cr	Significant decreases in NAA/Cr and Cho/Cr but no difference in Glx/Cr was found in glaucoma compared to healthy subjects in both the GCT and the striate area.

*BOLD, blood oxygenation level-dependent; LPZ, lesion projection zone; ROI, region of interest; VC, visual cortex; ACC, anterior cingulate cortex; L, left; R, right; ON, optic nerve*.

Functional activity resulted greatly reduced within primary visual cortex after visual input from the glaucomatous eye in all studies using fMRI both in POAG (Duncan et al., 2007a; Qing et al., 2010; El-Rafei et al., 2013; Lestak et al., 2014; Borges et al., 2015; Gerente et al., 2015; Murphy et al., 2016; Zhang et al., 2016; Jiang et al., 2017; Zhou et al., 2017b), NTG (El-Rafei et al., 2013; Lestak et al., 2014; Zhang et al., 2016) and PACG patients (Song et al., 2012).

Resting state fMRI (Rs-fMRI) is a tool for studying the human brain functional connectivity in resting state, without visual stimulation (Biswal et al., 2010). Voxel-wise degree centrality (DC) represents the number of connections for a given voxel and it, thus, quantifies the ability for information integration. The decreased connectivity was found in areas related to vision and also in other networks related to working memory and attention in POAG patients (Dai et al., 2013; Frezzotti et al., 2014, 2016; Chen et al., 2017; Giorgio et al., 2018). Decreased functional connectivity even without visual tasking was also observed in the bilateral visual cortices of PACG patients (Boucard et al., 2016).

Metabolic neuronal dysfunction was studied through quantification of certain biochemical compounds with proton MRS in two studies. The main brain metabolites studied by MRS included N-acetylaspartate (NAA), Choline (Cho), and Creatine (Cr). NAA is localized within neurons and a decreased in NAA concentration is a sign of neuronal loss. Choline is a marker of membrane integrity, it decreases when a damage of neurons occurred. Creatinine, implied in energy metabolism, should remain constant throughout the brain even in neurodegenerative disease. Its concentration is used to calculate metabolic ratio such as NAA:Cr and Cho:Cr. Results of the two studies using MRS were contradictory, Boucard et al (Boucard et al., 2007) found no significant differences in the brain metabolites between the patients and the control group. On the contrary, Zhang et al. (2013), found decreased brain metabolites which are marker for neuronal integrity in geniculo-calcarine and striate area of occipital pole.

## Discussion

Studies of glaucomatous degeneration in the brain were initially based on experiments conducted on monkey who were induced glaucoma. The studies identified degeneration at the lateral geniculate nucleus (LGN) and primary visual cortex (V1) in response to increased IOP and optic nerve damage (Yücel et al., 2000, 2001). These results were supported by the same results in post-mortem human studies that reported a significant neurodegeneration in optic nerve, LGN and visual cortex in a glaucoma patient (Gupta et al., 2006). The advent of non-invasive brain imaging techniques has led to an increase in studies investigating the involvement of the brain in glaucoma pathology. The detection of brain neurodegeneration in glaucomatous subjects could open new future frontiers. As an example, innovative brain imaging methods have been proposed in the early detection of the disease and in the evaluation of therapeutic efficacy of novel neuroprotective strategies (Brown et al., 2016). This systematic review is aimed at bringing together different results deriving from other research regarding brain involvement in patients with glaucoma.

### Integration of structural and functional results

Studying brain changes in glaucoma patients both structurally, functionally, and metabolically provides for more information.

#### White matter of visual pathway (NO, NT, OR)

Many studies using DTI demonstrated glaucomatous degeneration of the optic nerve (Garaci et al., 2009; Bolacchi et al., 2012; Nucci et al., 2012; Zhang et al., 2012; Wang et al., 2013; Omodaka et al., 2014), optic tract (Chen Z. et al., 2013; Frezzotti et al., 2014, 2016; Kaushik et al., 2014; Zhou et al., 2017a; Giorgio et al., 2018), and optic radiation (Garaci et al., 2009; El-Rafei et al., 2011, 2013; Engelhorn et al., 2011; Hernowo et al., 2011; Bolacchi et al., 2012; Chen Z. et al., 2013; Lu et al., 2013; Michelson et al., 2013; Frezzotti et al., 2014, 2016; Kaushik et al., 2014; Schoemann et al., 2014; Sidek et al., 2014; Gerente et al., 2015; Huang et al., 2015; Murai et al., 2015; Tellouck et al., 2016; Jiang et al., 2017; Zhou et al., 2017a; Giorgio et al., 2018). These findings are corroborated by other techniques, such as VBM. Subjects with glaucoma exhibited significant reductions in the volume of the visual pathway, including the optic nerves, chiasm, tracts, LGN, and optic radiations (Hernowo et al., 2011).

El-Rafei et al. demonstrated the ability of DTI- derived measures to differentiate between glaucomatous patients and healthy subjects but also between different glaucoma types (POAG and NTG) with an accuracy of 98.3% (El-Rafei et al., 2013).

According to Engelhorn's study (Engelhorn et al., 2011), rarefaction of optic radiation correlated both with the extent of optic nerve atrophy and with the extent of visual field defect. Thus, degeneration of optic radiation seems to be the result of anterograde transneuronal degeneration. Interestingly, the same study demonstrated a high prevalence (80%) of microangiopathic lesions within the optic radiation supporting the concept of retrograde degeneration of RGCs starting from the optic radiation. Moreover, axonal impairment of optic radiation studied with DTI demonstrated good correlation with cerebral glucose hypometabolism in the striate cortex of POAG patients (Murai et al., 2015).

#### Primary visual area

In regard to primary visual cortex (V1), loss of gray matter at SBM (Bolacchi et al., 2012; Yu et al., 2013, 2014, 2015; Wang et al., 2016) and VBM (Boucard et al., 2009; Li et al., 2012; Zikou et al., 2012; Chen W. W. et al., 2013; Frezzotti et al., 2014; Zhang et al., 2015; Giorgio et al., 2018) analysis is consistent with a reduction of function proved by fMRI studies (Duncan et al., 2007a; Qing et al., 2010; Song et al., 2012; El-Rafei et al., 2013; Borges et al., 2015; Gerente et al., 2015; Murphy et al., 2016; Zhang et al., 2016; Jiang et al., 2017; Zhou et al., 2017b).

Using VBM, Boucard et al. (2007) showed a retinotopic specific gray matter reduction in primary visual cortex. Indeed, POAG subjects showed a reduction in gray matter density mainly in the anterior part of occipital pole, while AMD patients showed a reduction located more posteriorly in occipital cortex, in correspondence with the location of the foveal representation in visual cortex.

Nevertheless, the study of Boucard et al. (2007) investigating the concentrations of metabolites such as N-acetylaspartate (NAA) in occipital pole using MRS, showed no alterations in patients with POAG compared to healthy subject. This could be explained with either absence of occipital degeneration or degeneration occurring with a slow rate, undetectable with MRS. The MRS method presents a limitation: it only measures reduced NAA levels when the degenerative process is currently taking place.

Interestingly, the deterioration of cortical activity and structure in V1 correlated with structural measurement of optic disc. Structural analysis of optic disc damage was conducted with scanning laser polarimetry with variable corneal compensation (GDx-VCC), confocal scanning laser ophthalmoscopy (SLO), and optical coherence tomography (OCT). In addition, functional activity of V1 is altered in a manner consistent with the loss of visual function (Duncan et al., 2007b), indicating that visual deprivation can have a potential effect on brain structure and function.

#### Higher visual areas and connection between visual areas

Many of the above-mentioned studies demonstrated the effect of glaucoma on higher visual areas, which are the secondary or associative areas of vision. Some of these studies showed a decreased functional activation after visual stimulation of the higher visual areas (Borges et al., 2015) and also a decreased cortical volume of associative areas (Bolacchi et al., 2012; Yu et al., 2013, 2014, 2015). The more plausible explanation of these changes is the transynaptic neurodegeneration. Other studies however found an increased functional activity in some higher visual areas (Jiang et al., 2017) and even an increased volume in some visual associative areas (Li et al., 2012; Chen W. W. et al., 2013; Williams et al., 2013; Jiang et al., 2017). Increased functional activity could be a result of reduced feedback signals from visual areas causing a reduction in GABA-mediated inhibitor signal (Chen et al., 2017). According to this latter hypothesis, visual area has an inhibition effect on some visual associative areas. Since the primary visual area is damaged in glaucoma, there is a reduction of inhibition that should cause a greater functional response to fellow eye input within the lesion projection zone. This was proved in murine (Crozier et al., 2007) and primate (Biswal et al., 2010) models but not in human eye (Boucard et al., 2007). Results of increased gray matter volume in visual association areas are more difficult to interpret. The increased volume t of these areas could be a sign of neuronal damage (microglia activation, neuronal swelling) or of increased cortical functionality and plasticity (increased arborization of dendrites and axons). The Jiang's study (Jiang et al., 2017) analyzed the same areas both in terms of volume with VBM and in terms of functional activity through BOLD level with the fMRI. Jiang et al. found that volume changes were not fully consistent with the regional blood flow changes; only frontal and parietal lobes have overlapped results. Increased volume in other areas without functional activation could be explained as the result of cell edema, sign of nerve damage.

Dai et al. (2013) found disrupted connectivity between the primary and higher visual cortex and between visual cortex and associative visual areas in the task-free state. Diminished connectivity between these areas may be associated with impairment in memory-related imagery, visual consolidation and integration of visual, auditory and tactile stimulation. In addition to areas of decreased connectivity, some brain regions of POAG patients showed increased connectivity with visual cortex. These changes could be related to recruitment to compensate areas of decreased activity or, alternatively, may be related to loss of inhibitor input.

Decreased connectivity in visual and working memory is confirmed by Frezzotti et al. study (Frezzotti et al., 2014) and it explains the impairment of object identification in POAG patients.

PACG patients share the same decreased visual information integration between the primary visual cortex and higher areas (Huang et al., 2015; Li et al., 2017).

#### Areas indirectly involved in the visual pathway

Several of the studies that this paper is based on reported alterations in areas not directly involved in the visual pathway. A study which measured the brain connectivity with the rs-fMRI in PACG patients (Cai et al., 2015) showed the increased connectivity in the anterior cingulate cortex (ACC) and the caudate, a component of the basal ganglia. The ACC is responsible for cognition and emotional control; its dysregulation in PACG patients could be caused by the higher prevalence of anxiety and depression in glaucomatous patients (Agorastos et al., 2013), even higher in PACG (Kong et al., 2015). The increased connectivity in the caudate is explained as a response to altered proprioception and somatosensory processing.

Decreased spontaneous activity in the claustrum and paracentral lobule lobe was observed in PACG patients using the same technique (rs-fMRI) (Chen et al., 2017). These finding may indicate impairment of the visuospatial function; the results of Zikou et al corroborate this hypothesis for the same reason (Zikou et al., 2012).

Alterations in extra-visual pathway areas are also demonstrated with structural examinations. A recent study using DTI (Boucard et al., 2016) demonstrated the degeneration of the corpus callosum in a cohort of normal tension glaucoma. This finding suggests the presence of neurodegeneration of the brain beyond what can be explained on the basis of propagated retinal and pre-geniculate damage. It is arduous to apply this conclusions to POAG patients; it is more reasonable that NTG has a different profile of neuronal cell death. Another area which showed volumetric alteration in glaucomatous patients was the amygdala that plays a role in the control of emotions (Wang et al., 2016).

In Frezzotti's study POAG patients showed gray matter atrophy in regions involved in cognitive processing (Frezzotti et al., 2014). In addition, several areas showed alteration in both Alzheimer disease and glaucoma: the hippocampus, involved in the memory process, the frontoorbital cortex (implied in the decision-making) and superior parietal lobule (special orientation).

While glaucoma normally manifest as a damage in main cerebral visual areas, there are also alterations possibly related to glaucoma in areas of the brain that are less known of. These parts of the brain for example are involved in other functions, emotional, decision making and memory.

### Possible causes of cortical changes in glaucoma patients

Mechanisms involved in cortical changes of glaucomatous patients remain debated. Also, the timing of the neurodegenerative cortical process is still to be unanimously ascertained. The main question is whether the cortical changes precede or follow the RGC neurodegeneration. Many studies supported the thesis that glaucomatous degeneration of the posterior visual pathway is a consequence of anterograde trans-synaptic diffusion of death signals triggered by RGC degeneration (Calkins and Horner, 2012). Bolacchi et al. (2012) analyzed the properties of the intraorbital optic nerve at two different levels: proximal and distal to the optic nerve head using the DTI. The study results indicate at early disease stage a significant decrease in connectivity in the proximal segment with respect to the distal site. Furthermore, reduced brain volume in visual-related cortex was found only in advanced stage glaucoma but not in early stage patients (Li et al., 2012). Functional analysis through fMRI provided a detailed analysis of layer-specific neuronal signals in the subcortical visual nuclei (lateral geniculate nucleus and superior colliculus) and visual cortex. It emerges that early-stage glaucoma causes selective functional loss to large cells (responsive to M stimulus) in the lateral geniculate nucleus and superior colliculus, but not in the visual area (Zhang et al., 2016).

On the other hand, similarities between glaucoma and other neuro-degenerative diseases (including Alzheimer's disease) suggest a certain grade of retrograde trans-synaptic degeneration as well. A demonstration of this theory is the presence of damage also in areas not directly involved in the visual pathway (Cai et al., 2015; Boucard et al., 2016). In addition, functional changes in brain connectivity (Frezzotti et al., 2016; Murphy et al., 2016) together with reduced brain volume were found in early stage glaucoma, even before visual field damage (Murphy et al., 2016).

Vascular changes also have a possible role in the physiopathology as demonstrated by ischemic microinfarcts in the optic radiation and other cortical regions (Boucard et al., 2016).

### Correlation of structural and functional parameters with clinical severity

Many studies investigated the correlation between structural, functional and metabolic brain parameters and ophthalmic measurement such as cup-disc ratio, retinal nerve fiber layer thickness and visual field parameters (Hernowo et al., 2011; Nucci et al., 2012; Chen Z. et al., 2013; Michelson et al., 2013; Sidek et al., 2014; Cai et al., 2015; Gerente et al., 2015; Chen et al., 2017; Li et al., 2017). Many studies found a positive correlation with all these parameters, revealing that visual pathway damage correlates with clinical severity in glaucoma (Nucci et al., 2012; Chen Z. et al., 2013; Michelson et al., 2013; Sidek et al., 2014; Cai et al., 2015; Gerente et al., 2015; Chen et al., 2017).

The relationship with loss of visual field has important implications. In glaucoma a loss of ~40−60% of the ganglion cells is already present when visual field defects are detectable by perimetry (Quigley et al., 1989; Harwerth et al., 1999, 2005; Kerrigan-Baumrind et al., 2000; Rolle et al., 2016). Structural neuroimaging techniques, such as DTI, are able to detect axonal injury of visual pathway at an early stage, even before visible visual field defect.

Moreover, a decreased cortical activity in the primary visual cortex is detected with fMRI in the cortical area corresponding to the central normal field (Qing et al., 2010). As previously reported, a big amount of RGC is lost before any abnormality in automated visual field testing is detectable. The remaining RGC undergo structural reorganization which consist in somata and dendritic arbors expansions. Consequently, cortical visual receptive filed increases in size. Thus, even if the central visual field is normal, the retina input is already reduced and the activity in visual cortex is reduced as well.

It is not surprising, that many authors propose neuroimaging techniques as a tool for earlier diagnosis of glaucoma in the future.

In regard to influence of glaucoma severity on brain changes, it is somehow difficult to provide a systematic analysis mainly because of differences in classification and parameters used to divide patients. Nevertheless, while functional connectivity was proved to be already altered in early POAG in both visual and nonvisual systems (Frezzotti et al., 2016; Zhang et al., 2016), structural changes (mainly gray matter atrophy) was unanimously found in more advanced/severe POAG, but not in all early POAG analyzed (Li et al., 2012; Zhang et al., 2015). Studies investigating cortical geometry in mild and severe POAG demonstrated a progressively aggravated degeneration with the increasing severity of disease. Cortical degeneration occurs not only in visual cortex (both V1 and V2), but also in the middle temporal visual area (V5/MT+), which is thought to be specialized for processing motion (Yu et al., 2014, 2015).

Overall, structural, functional, and metabolic brain changes show a good correlation with clinical measurement of severity.

### Neuronal plasticity

Many human studies including those of this review have suggested that even during adulthood the visual cortex maintains the ability to structurally and functionally reorganize (Rosa et al., 2013). Visual cortical plasticity was also proved in healthy subjects after visual training (Berry and Nedivi, 2016). Neuroplasticity can be both structural, which implies changes in axons, and functional (Rosa et al., 2013). Functional reorganization is related to the unmasking of intracortical feedback to early visual cortex from higher visual areas (Crozier et al., 2007; Biswal et al., 2010).

Postoperative enhancement in neuronal connectivity was demonstrated in a group of PACG patients 3 months after surgery with normalization of IOP (Cai et al., 2015). The areas that showed a better connectivity were visual cortex, primary, sensory, and supplementary motor areas Further studies (Li et al., 2017) demonstrated functional restoration after glaucomatous surgery and IOP normalization. One possible explanation for this result is the post-operative plasticity in functional networks. These changes can occur 3 months after the surgery, and it is possible that they are due to restoration process. Li et al. found a decreased connectivity of the visual area with the resting state-fMRI after surgery and IOP normalization (Li et al., 2017). The decreased connectivity of the visual area reflects the decreased compensation of other brain areas following the reduction of IOP and improving vision.

On the contrary, some authors suggested a remarkable level of stability within the adult primary and extrastriate visual cortex, without any evidence of neurodegeneration affecting cells receiving visual information of fellow eye in patients with unilateral glaucoma (Borges et al., 2015).

### Limits and suggestion for future studies

Our study suffers from several limitations mainly due to limitations associated with experimental design and available tools of studies. First, different brain atlases were used to annotate localization of cortical areas. Moreover, in regard to the fMRI studies, differences in type, frequency, size, brightness, and color of stimuli are present in different studies. Thus, cortical activity is certainly influenced by the stimulus which causes conclusions of studies to be partially conflicting with each other. Another limitation consists in the heterogeneity of age of patients, severity of damage and duration of disease between the studies.

Some suggestion for future studies include a consistent number of both glaucomatous and healthy subject, division of glaucomatous patients in severity group through well-established classification (i.e., Hodapp criteria), application of structural, functional and metabolic methods. Correspondence of structural and functional-metabolic abnormalities gives strength to results.

### Therapeutic perspectives

The identification of neurodegeneration in the visual centers of the brain has important implications for the treatment of glaucoma. Emerging therapeutic interventions, such as RGC transplantation and artificial retinal implants (Mathieson et al., 2012; Venugopalan et al., 2016; Jutley et al., 2017; Sena and Lindsley, 2017), need to consider the degree of neurodegeneration in the posterior visual pathway as a confounding factor in the efficacy of therapeutic interventions. Glaucomatous neurodegeneration in the brain also has implications for assessing the efficacy of neuroprotection, suggesting that the focus of these therapies should be extended to include the whole brain. Furthermore, abnormalities of the brain should be included as endpoints in studies of therapeutic efficacy in glaucoma trials.

## Conclusion

Systematic review yields the advantage of aggregating information and more robust estimates than individual studies. This approach can be particularly useful when evaluating new procedures with relatively small studies. Brain changes in glaucomatous subjects have been proved with autoptic and experimental studies in the past. Recently, the advent of non-invasive neuroimaging has led to a growth in studies investigating the brain damage of glaucomatous patients.

The results of this meta-analysis show functional and structural changes throughout the visual and nonvisual pathway in glaucomatous patients. These changes correlate with clinical findings and severity of the disease. Entity and mechanism of cortical reorganization are still not completely clear.

This review contributes to the better understanding of brain abnormalities in glaucoma. The results may stimulate further speculation about brain plasticity at a later age and therapeutic strategies, such as the prevention of cortical degeneration in patients with glaucoma.

## Author contributions

RN conception and design of study. LD acquisition and analysis of data. LD and TR drafting the manuscript. RN, LD, and TR revising the manuscript critically for important intellectual content. RN, LD, and TR approval of the version of the manuscript to be published.

### Conflict of interest statement

The authors declare that the research was conducted in the absence of any commercial or financial relationships that could be construed as a potential conflict of interest.
