# Supplementary material for: Changes of Visual Pathway and Brain Connectivity in Glaucoma: A Systematic Review
Source: Front Neurosci. 2018 May 29;12:363. doi: 10.3389/fnins.2018.00363 (PMC5986964; doi:10.3389/fnins.2018.00363)
Supplement: Supplementary file 1 [file Data_Sheet_1.DOCX]

Supplementary Material

Changes of visual pathway and brain connectivity in glaucoma: a systematic review

Raffaele Nuzzi*, Laura Dallorto, Teresa Rolle

*** Correspondence:** Raffaele Nuzzi: prof.nuzzi_raffaele@hotmail.it

# MEDLINE search strategy

((“Functional magnetic resonance” OR “Functional magnetic resonance imaging” OR fMRI) OR (“resting state functional magnetic resonance” OR “resting state functional magnetic resonance imaging” OR rs-fMRI) OR (MRS OR “magnetic resonance spectroscopy”) OR (SBM OR “surface-based morphometry” OR “surface-based analysis”) OR (VMB OR “Voxel based morphometry” OR “Voxel based analysis”) OR ( DTI OR “ diffusion tensor MRI”))

AND

(“grey matter” OR cerebr* OR cortex OR (visual AND (pathways OR cortex OR cortic* OR cerebr*)) AND (glaucoma OR (glaucom* AND disease) OR POAG OR NTG OR PACG)
